# Jumonji domain‐containing protein 6 protein and its role in cancer

**DOI:** 10.1111/cpr.12747

**Published:** 2020-01-21

**Authors:** Jing Yang, Siyuan Chen, Yanfei Yang, Xuelei Ma, Bin Shao, Shengyong Yang, Yuquan Wei, Xiawei Wei

**Affiliations:** ^1^ Laboratory of Aging Research and Nanotoxicology State Key Laboratory of Biotherapy and Cancer Center National Clinical Research Center for Geriatrics West China Hospital Sichuan University Chengdu Sichuan China; ^2^ State Key Laboratory of Oral Disease West China Hospital of Stomatology Sichuan University Chengdu Sichuan China

**Keywords:** cancer, epigenetics, JMJD6 protein, jumonji domain‐containing histone demethylases

## Abstract

The jumonji domain‐containing protein 6 (JMJD6) is a Fe(II)‐ and 2‐oxoglutarate (2OG)‐dependent oxygenase that catalyses lysine hydroxylation and arginine demethylation of histone and non‐histone peptides. Recently, the intrinsic tyrosine kinase activity of JMJD6 has also been reported. The JMJD6 has been implicated in embryonic development, cellular proliferation and migration, self‐tolerance induction in the thymus, and adipocyte differentiation. Not surprisingly, abnormal expression of JMJD6 may contribute to the development of many diseases, such as neuropathic pain, foot‐and‐mouth disease, gestational diabetes mellitus, hepatitis C and various types of cancer. In the present review, we summarized the structure and functions of JMJD6, with particular emphasis on the role of JMJD6 in cancer progression.

## 1 INTRODUCTION

The term “epigenetics” was first conceived by Conrad H. Waddington in the early 1940s to describe heritable changes in gene expression without alteration in DNA sequences.^1^ Deregulation of epigenetic processes leads to altered gene functions and a wide variety of pathologies, such as autoimmune diseases, metabolic diseases, neurological disorders and cancer.^2^, ^3^, ^4^ The key processes responsible for epigenetic regulation include DNA methylation, histone modification, nucleosome remodelling and alterations in non‐coding RNA profiles.^1^, ^2^ Histone methylation and hydroxylation, involving a wide range of epigenetic processes, have attracted considerable attention in the past decade.^5^

The jumonji C (JmjC) containing family of proteins are mainly composed of histone‐modifying enzymes, which are Fe(II)‐ and 2‐oxoglutarate (2OG)‐dependent oxygenases.^6^ The jumonji domain‐containing protein 6 (JMJD6), a member of the JmjC domain‐containing proteins, was originally identified as a phosphatidylserine receptor (PSR, Ptdsr) on cell surface.^7^ Subsequent studies demonstrated that JMJD6 is located in the nucleus, and has arginine demethylase and lysyl hydroxylase activities in histone and non‐histone proteins.^8^, ^9^ In addition, JMJD6 can also function as a tyrosine kinase of histone.^10^ There is growing evidence indicating the functions of JMJD6 at the transcriptional, splicing, posttranscriptional and biochemical levels, although the precise molecular mechanisms are not yet clear.^8^, ^11^, ^12^ JMJD6 promotes cell proliferation and migration in vitro, and accelerates tumour growth in vivo.^13^, ^14^ Overexpression of JMJD6 is correlated with advanced clinicopathological stage, increased aggressiveness and poor survival.^12^, ^15^, ^16^, ^17^

Here, we summarized the structure and functions of JMJD6 based on relevant basic researches. In particular, we focused on the role of JMJD6 in cancer progression and candidate mechanisms in order to highlight that JMJD6 may represent an attractive target for a new generation of anticancer drugs.

## 2 THE JMJD6 PROTEIN AND ITS STRUCTURE

### 2.1 From phosphatidylserine receptor to JMJD6

In 2000, JMJD6 was originally misassigned as a PSR expressed on the surface of macrophages, fibroblasts and epithelial cells.^7^, ^18^ Later studies reported the phosphatidylserine‐mediated clearance of apoptotic cells initiated by homologue of JMJD6 in *Caenorhabditis elegans* and zebrafish.^19^, ^20^ However, subsequent studies suggested that the function was incorrectly assigned and that the protein is predominantly located in cellular nucleus. In JMJD6‐deficient mice, the elimination of JMJD6 function leads to serious defects in the morphology of multiple organs and neonatal lethality, which cannot be explained by impaired apoptotic cell clearance.^9^ Therefore, JMJD6 was first demonstrated to be essential for the development, differentiation and maturation of multiple tissues during embryogenesis but not for apoptotic cells removal.^9^

In contrast to the proposed localization on cellular surface, a later study demonstrated that protein encoded by the JMJD6 cDNA is localized in the nucleus both in transfected cells and in cells expressing endogenous JMJD6 mRNA.^21^ Meanwhile, by cloning the homologous genes in Hydra, another study suggested that JMJD6 is a nuclear 2OG‐and Fe(II)‐dependent oxygenase that is capable of modifying nuclear proteins.^22^ Owning to this discovery and subsequent confirmation, the PSR was renamed to JMJD6.^8^, ^22^, ^23^, ^24^ JMJD6 exists in both cytoplasm and nucleus of MeWo cells; in the cytoplasm, JMJD6 presents as a soluble protein and associates with intracellular vesicles.^25^ JMJD6 has also been reported to be a secreted protein that can be detected in extracellular matrix.^25^

### 2.2 Structure of JMJD6

**Figure 1:**
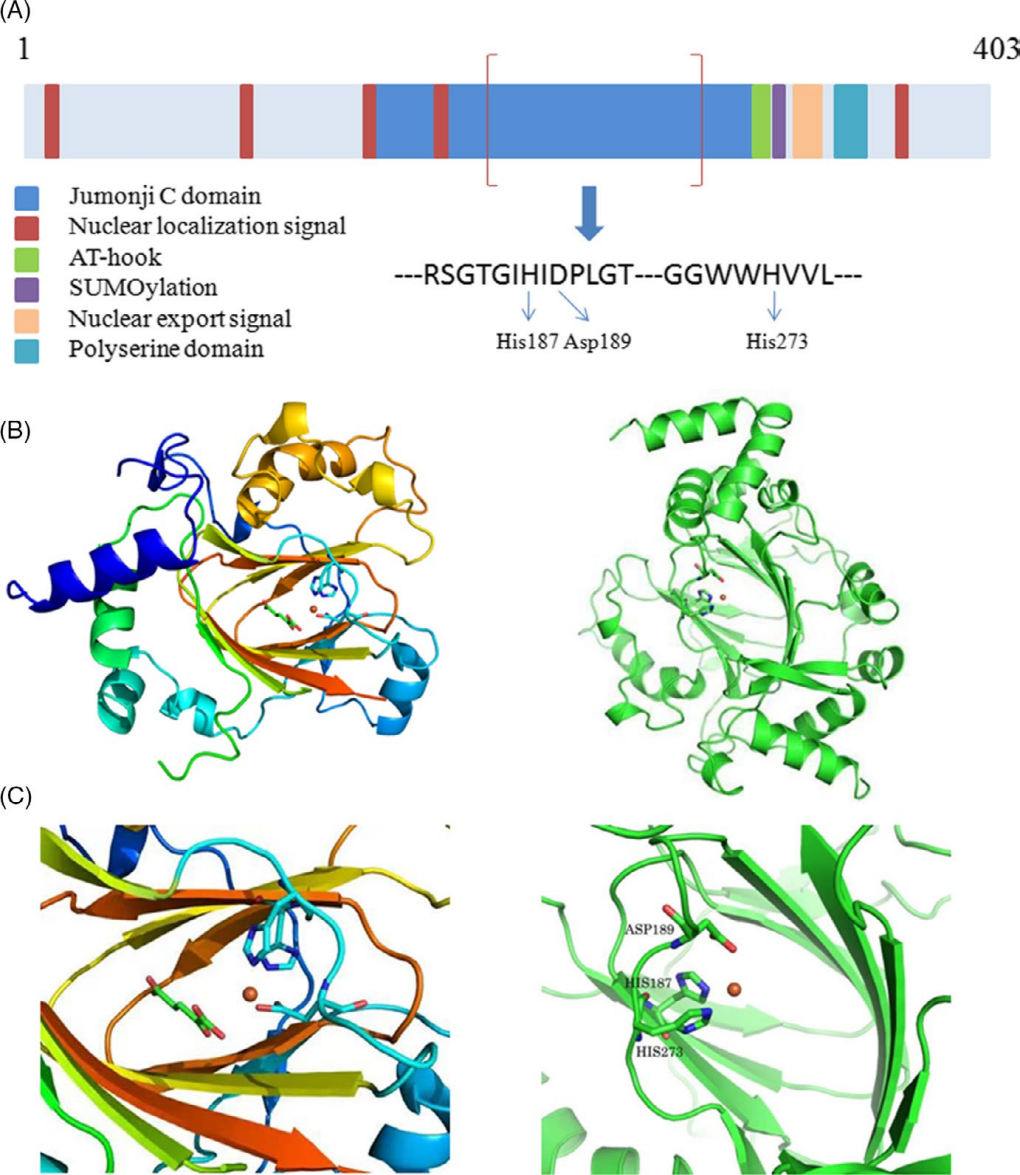
A, Diagram of the domain structure of Jumonji domain‐containing protein 6 (JMJD6). B, Three‐dimensional cartoon depicting the structure of JMJD6. C, The detailed structure of the HXD/E(X)nH motif in JmjC Domain, which is essential for the enzymatic activities of JMJD6. Conserved sequence motifs of JMJD6 protein include the following: a central Jumonji C domain (JmjC domain) (residues Pro141 to Gln286), five nuclear localization signals (NLSs), a DNA binding domain (AT‐hook) (residues Lys300 to Ser309), a putative SUMOylation site (Leu316 to Glu319) and a polyserine (polyS) region. His187, Asp189 and His273 are Fe (II) complexing residues

The results of sequence analysis demonstrated that JMJD6 contains a JmjC domain, which is highly conserved in proteins from eukaryotes to bacteria (Figure 1).^21^, ^26^, ^27^ The common core protein structural fold of all 2OG‐dependent oxygenases comprises the typical cupin or double‐stranded β‐helix fold (DSBH) (formed by 8 β‐strands within the JmjC domain) surrounded by characteristic secondary structure elements.^28^ Crystallographic studies confirmed the structure of DSBH; the DSBH fold forms a barrel‐like structure with two β‐sheets, and the Fe(II) binding site of the catalytic centre is located at the opening end of the barrel. The metal is most commonly bound by the side chain of three residues (His187, Asp189 and His273), which form an HXD/E(X)nH motif and are essential for the enzymatic activities of JMJD6.^24^, ^29^, ^30^, ^31^

In addition to the JmjC domain, five nuclear localization signals (NLSs), a nuclear export signal (NES), a DNA binding domain (AT‐hook) (residues Lys300 to Ser309) and a putative SUMOylation site (Leu316 to Glu319) are the conserved sequence motifs of JMJD6 protein (Figure 1).^22^, ^26^, ^27^, ^32^ Examinations of JMJD6 amino acid sequence showed the presence of five functional NLSs that can target JMJD6 to the nuclei either alone or in concert.^21^, ^22^ Two of the five NLSs overlap with the JmjC domain and may not be topologically accessible in vivo.^21^ The AT‐hook was initially described as a DNA binding motif; however, JMJD6 binds efficiently to single‐stranded RNA, but does not bind to DNA.^33^, ^34^, ^35^ JMJD6 was suggested to be a type of non‐canonical AT‐hook‐like domain protein.^35^ Using the CBS‐prediction service, a putative SUMOylation site (probability score 92%) was identified in the JMJD6 protein, and the SUMOylation site might be used to regulate its interactions with other proteins.^26^ Furthermore, three‐dimensional structural model of JMJD6 protein indicates that the NLSs, the AT‐hook and the SUMOylation site may be accessible for interacting proteins.^26^

Jumonji domain‐containing protein 6 has a polyserine (polyS) region at its C‐terminus. The polyS region is highly conserved and comprises 16 serine residues interrupted by 4 aspartate residues (Ser340‐Ser359) (Figure 1).^32^ This C‐terminal polyS is missing in JMJD6 splice variants.^26^ In some bacterial extracellular modular carbohydrate degrading enzymes, the polyS region was suggested to be a flexible linker connecting substrate and binding enzymatic domains.^36^ A cell‐based study demonstrated that the polyS region of JMJD6 protein participates in the bidirectional nucleoplasmic‐nucleolar shuttling of JMJD6.^32^ The presence/absence of the polyS region regulates the subnuclear localization of JMJD6 protein. In the JMJD6 protein, the polyS domain may have a regulatory influence on its oligomeric structure. Transmission electron microscopy (TEM) studies indicated that the structure of JMJD6 oligomer depends on the presence of the polyS domain; JMJD6 lacking the polyS domain forms a filamentous structure, while JMJD6 with complete polyS domain forms a ring‐shaped overall oligomeric structure.^32^

JMJD6 can exist in monomeric and larger oligomeric forms.^37^ Several studies showed that JMJD6 adopts an oligomeric form in solution. However, by Western blot analysis of full‐length recombinant JMJD6, both monomeric and oligomeric forms were detected in solution, and the oligomers correspond to apparent trimeric, pentameric and larger oligomeric forms.^31^, ^38^, ^39^, ^40^ The existence of JMJD6 oligomerizes in cells was confirmed by co‐immunoprecipitation and fluorescence two‐hybrid assays.^32^, ^40^

## 3 THE ENZYMATIC ACTIVITIES OF JMJD6

**Figure 2:**
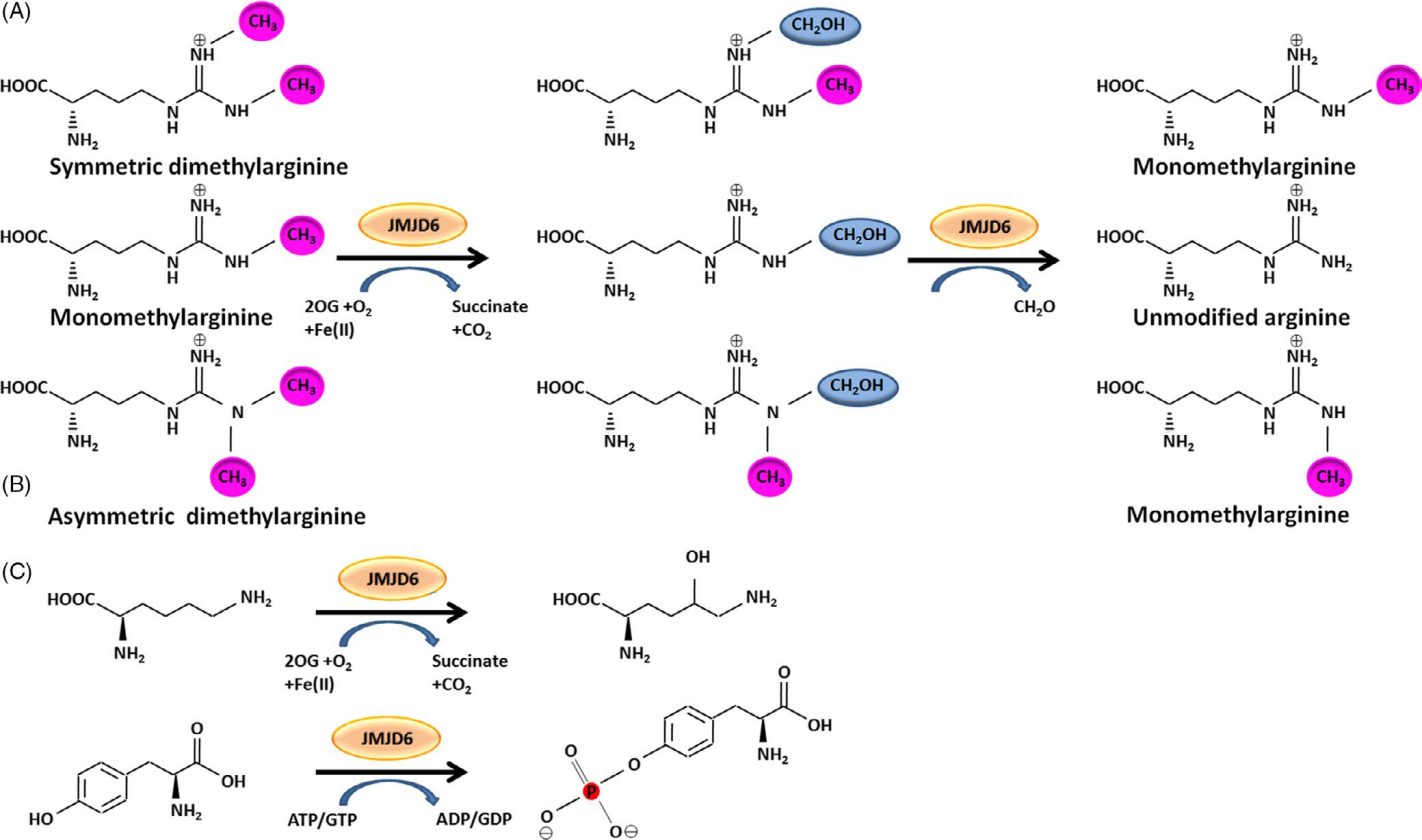
JMJD6 functions as arginine demethylase (A), lysyl hydroxylase (B) and tyrosine kinase (C). A, Demethylation reactions of symmetric dimethylarginine, monomethylarginine and asymmetric dimethylarginine catalysed by JMJD6. In the first step, JMJD6 hydroxylates the methyl group consuming oxoglutarate (2OG), and in the second step, a deformylation reaction produces formaldehyde (CH2O) to form an unmodified arginine. B, JMJD6 catalyses lysine hydroxylation. C, JMJD6 phosphorylates tyrosine using both guanosine triphosphate (GTP) and adenosine triphosphate (ATP) as phosphate donors

So far, the enzymatic activities are considered to be the most important characteristics of JMJD6. JMJD6 has been reported to possess arginine demethylase and lysyl hydroxylase activities for a long time (Figure 2A,B). Recently, the intrinsic tyrosine kinase activity of JMJD6 was also observed. JMJD6 phosphorylates Y39 of histone H2A.X using both guanosine triphosphate (GTP) and adenosine triphosphate (ATP) as phosphate donors and regulates the expression of autophagy‐related genes (Figure 2C).^10^ Unlike any known kinase protein, JmjC domain and the polyS domain are required for the kinase function of JMJD6. Overall, JMJD6 is likely to have many potential catalytic activities, but the long‐standing controversies surrounding the enzymatic spectrum of JMJD6 should be addressed through more in‐depth experiments. Here, we mainly summarized the two enzymatic actions of JMJD6: hydroxylation and demethylation.

### 3.1 JMJD6 as an arginine demethylase

One of the proposed functions of JMJD6 is catalysing arginine demethylation (Figure 2A). This reaction depends on the presence of cofactors, including Fe (II) and 2OG.^37^ Arginine methylation occurs in many proteins involved in various cellular functions. Among them, histones have long been known to be substrates for methylation.^41^ Methylation of histone plays an important role in the regulation of transcription, genome integrity and epigenetics.^5^ In mammals, methylation of histone arginine is typically found on several residues, including residues 2, 8, 17 and 26 of histone H3 (H3R2, H3R8, H3R17, H3R26) and residue 3 of histone H4 (H4R3).^41^, ^42^ In 2007, JMJD6 was first experimentally demonstrated to function as a dioxygenase.^8^ Notably, JMJD6 demethylates monomethylarginine and symmetric and asymmetric dimethylarginine residues (Figure 2A). Recent studies found that JMJD6 also targets arginine residues of non‐histone proteins for demethylation, including RNA helicase A, oestrogen receptor α (ERα), tumour necrosis factor receptor‐associated factor 6 (TRAF6), the transcription factor PAX3 and heat‐shock protein 70 (HSP70).^43^, ^44^, ^45^, ^46^, ^47^, ^48^

The role of JMJD6 as a histone arginine demethylase remains controversial. On the one hand, evidence that JMJD6 directly demethylates proteins is still absent, and therefore, we cannot rule out that JMJD6 may indirectly affect the demethylation of these proteins. On the other hand, several studies reported that histone arginine demethylation activity of JMJD6 was not observed in their study.^23^, ^49^, ^50^ JMJD6 was incubated with arginine–serine‐rich (RS) domain (arginine‐rich sequences present in this domains) in the presence of oxygen, Fe(II) and 2OG, and subsequently analysed by means of matrix‐assisted laser desorption/ionization (MALDI) mass spectrometry (MS).^23^ The results showed that JMJD6 cannot produce demethylated arginine histone H4 and H3 fragment peptides.^23^ In another study, JMJD6 silencing in endothelial cells was shown to not affect arginine methylation at H4R3.^50^ In addition, a study on the crystal structure of JMJD6 also doubts on its ability to demethylate arginine residues based on the structural data.^49^ Even so, arginine demethylation catalysed by JMJD6 cannot be ruled out and further studies are expected.

### 3.2 JMJD6 as a lysyl hydroxylase

In addition to its arginine demethylase activity, JMJD6 also has strong lysyl hydroxylase activity (Figure 2B).^23^, ^51^, ^52^ After incubation of U2 small nuclear ribonucleoprotein auxiliary factor 65‐kilodalton subunit (U2AF65) with JMJD6, 2OG and iron, the results of liquid chromatography‐mass spectrometry (LC‐MS)/MS analysis showed that U2AF65 is a substrate of JMJD6 and JMJD6 executes lysine‐specific hydroxylation of U2AF65 (from HeLa cells) at lysine K15 (hydroxylated:unhydroxylated, 1:100) and K276 (hydroxylated:unhydroxylated, 1:250) residues.^23^ Moreover, in HeLa cells, JMJD6 overexpression results in increased hydroxylation of U2AF65.^23^ No evidence of hydroxylation of lysyl residues in endogenous histones (H2A, H2B, H3 and H4) was accrued in this study.^23^ However, in 2013, another study developed an alternative method, namely amino acid composition analysis, to detect 5‐hydroxylation of histone lysyl residues.^51^ This study reported that JMJD6 can hydroxylate multiple lysine residues of histone H3 and H4.^51^ It indicates that in addition to the only known lysyl hydroxylases, the procollagen lysyl hydroxylase (PLOD enzymes), JMJD6 also functions as a specialized lysyl hydroxylase.

### 3.3 Regulation of JMJD6 functions

The activity of JMJD6 is regulated by hypoxia, iron and 2OG availability.^31^ The upregulation of JMJD6 can be induced by hypoxia.^53^ The hypoxia‐inducible factor (HIF) hydroxylases are the major regulators of the hypoxia response, probably in animals ranging from nematode worms to man.^54^ Hypoxia can upregulate metastasis‐associated lung adenocarcinoma transcript 1 (MALAT1), a long non‐coding RNA associated with cancer progression and metastasis.^55^ JMJD6 can be positively regulated by MALAT1 through MALAT1/miR‐125/JMJD6 axis. Furthermore, tricarboxylic acid (TCA) cycle intermediates, including succinate, fumarate and succinate, can inhibit the activities of 2OG oxygenases.^48^, ^56^

## 4 JMJD6 IN CANCER PROGRESSION

Abnormal expression of JMJD6 may contribute to the development of many diseases, such as neuropathic pain, foot‐and‐mouth disease, gestational diabetes mellitus, hepatitis C and various types of cancer.^12^, ^57^, ^58^, ^59^, ^60^, ^61^ Here, we summarized the role of JMJD6 in the progression of several types of cancer (Table 1).

**Table 1:** The role of JMJD6 in different types of cancer

Cancer type	Findings from in vitro and in vivo studies	Findings from clinical data	References
Breast cancer			10, 12, 14, 23, 62
ER− breast cancer	JMJD6 is associated with increased cell proliferation, migration, invasion and metastases	JMJD6 expression is positively correlated with histological grade, age, LN metastasis, tumour size and advanced TNM stage. High level of JMJD6 may/not indicates poor survival (*different conclusions are reported*)	
ER+ breast cancer	JMJD6 promotes/inhibits proliferation and migration of MCF‐7 cells (*contradictory conclusions are reported*)	Expression of JMJD6 in ER+ tumours is slightly but significantly lower than ER− tumours. JMJD6 is highly expressed in more aggressive and advanced tumours. High JMJD6 expressers have poorer outcomes than low expressers	
Melanoma	JMJD6 facilitates proliferation and invasion of melanoma cells in vitro, and promotes growth and metastasis of melanoma in vivo. JMJD6 enhances blood vessel formation	JMJD6 expression is increased in melanoma. At later stages of melanoma progression, JMJD6 level is elevated. High level of JMJD6 is associated with unfavourable prognosis	63, 64
Oral cancer	JMJD6 is enriched in cancer stem cells. JMJD6 promotes cancer stem cell properties, and knock‐down of JMJD6 suppresses stem‐like property of OSCC	Expression level of JMJD6 is higher in carcinoma tissues than in normal tissues	66
Lung adenocarcinoma	Acetylation of HOXB9 at lysine 27 decreases its ability to promote the migration and growth of lung cancer cells in mice through suppressing the transcription of JMJD6	JMJD6 mRNA and protein are significantly increased in human lung adenocarcinoma specimens. The level of JMJD6 is significantly associated with clinical parameters. The survival of patients with high JMJD6 expression is poorer than those with low JMJD6 expression	71, 72
Glioblastoma	Inhibiting JMJD6 with shRNA could improve survival in the orthotopic xenograft mouse model of glioblastoma, but could not alter cell growth and survival in vitro	JMJD6 mRNA and protein are significantly elevated in human gliomas tissues and are increased with tumour grade	73, 74
Hepatocellular carcinoma	JMJD6 promotes proliferation and migration of HCC cell lines. JMJD6 regulates cell cycle and apoptosis progression of HCC cell lines	JMJD6 is significantly correlated with tumour grade and TNM stage. High JMJD6 expression in tumour tissue is indicative of poor prognosis	17
Colon carcinoma	JMJD6 increases the percentage of cells in the G1 phase, promotes cell apoptosis and sensitizes cells to DNA damaging agents. JMJD6 promotes cellular proliferation and tumorigenesis in vivo	JMJD6 protein is significantly increased in colon adenocarcinomas. High level of JMJD6 expression is correlated with increased invasiveness, poor differentiation, lymph node metastases and advanced stage. JMJD6 is associated with worse prognosis	16
Ovarian cancer	Inhibition of JMJD6 suppresses cellular proliferation and migration, and promotes apoptosis of ovarian cancer cell lines. JMJD6 inhibitor exhibits powerful therapeutic effects on ovarian cancer in vivo	JMJD6 is highly expressed in 61.64% of 146 ovarian cancer patients. High expression of JMJD6 is significantly associated with age, clinical stage, pT status and pN status of the ovarian cancer patients. High level of JMJD6 indicates high risk of disease progression and death	75
Neuroglioma	JMJD6 expression is increased notably in neuroglioma stem cells than other neuroglioma cells. JMJD6 promotes proliferation, migration and invasion of neuroglioma stem cells	—	13

Abbreviations: HCC, hepatocellular carcinoma; JMJD6, jumonji domain‐containing protein 6; LN, lymph node; OSCC, oral squamous cell carcinoma; pN status, pathological node status; pT status, pathological tumour status; TNBC, triple‐negative breast cancer; TNM, tumour node metastasis.

### 4.1 Breast cancer

In triple‐negative breast cancer cell lines, oestrogen‐induced breast cancer cells and MMTV‐Myc mammary tumour cells, in vitro and in vivo experiments indicated that high level of JMJD6 leads to increased cell proliferation, migration, invasion and metastases.^12^, ^62^ In MCF7 breast cancer cell line, Poulard et al reported that JMJD6 promotes proliferation and migration in vitro and tumour growth in vivo, whereas Lee et al reported opposite findings.^14^ Combined inhibition of JMJD6 kinase activity and autophagy is an effective therapeutic strategy for triple‐negative breast cancer.^10^ In mice injected with breast carcinoma cells, treatment with P4E11, a monoclonal antibody specific for JMJD6, can reduce fibrosis at the primary tumour and metastatic burden by blockading the interaction of JMJD6 with collagen I, which is also confirmed in xenograft mouse model of ovarian cancer.^25^

In breast cancer samples from patients, the expression of JMJD6 is different in different breast cancer subtypes: JMJD6 expression in ER‐positive (ER+) tumours is slightly but significantly lower than in ER‐negative (ER−) tumours (JMJD6 expression is consistently associated with ER− disease); the expression of JMJD6 is highest in Claudin‐low and basal subtypes followed by HER2‐enriched and luminal B subtypes, and lowest in luminal A subtype.^12^ In both ER+ and ER− breast cancer patients, elevated expression of JMJD6 is positively associated with histological grade, age, lymph node metastasis, tumour size and advanced tumour node metastasis (TNM).^12^, ^62^ Unfavourable survival was observed in high JMJD6 expressers with ER+ breast cancer.^10^, ^62^ However, in ER− breast cancer patients, several studies have reported that there is no significant correlation between JMJD6 level and survival, probably due to the high level of JMJD6 expression in ER− breast cancer.^12^, ^62^ Furthermore, the expressions of JMJD6 in cancer tissues and paired adjacent tissues are different in different breast cancer subtypes; JMJD6 levels in cancer tissues are higher than in adjacent matched tissues in 90% patients with triple‐negative breast cancer, but this ratio is 10% in patients with other breast cancer subtypes. Taken together, the role of JMJD6 in promoting breast cancer progression has been established, although its role in different subtypes of breast cancer may not be identical.

### 4.2 Melanoma

Jumonji domain‐containing protein 6 regulates melanogenesis in melanoma cells because overexpression of JMJD6 promotes the expression of microphthalmia‐associated transcription factor (MITF), a master regulator of melanogenesis.^63^ JMJD6 facilitates multiple cellular processes, including proliferation and invasion of melanoma cells in vitro, and promotes growth and metastasis of melanoma in vivo.^63^ At later stages of melanoma development in zebrafish, the expression of JMJD6 is elevated.^64^ Furthermore, JMJD6 is capable of enhancing blood vessel formation in melanoma.^63^

In human melanoma tissues, JMJD6 expressions were increased in both primary and metastatic melanomas than normal tissues, with higher expression of JMJD6 in metastatic melanoma.^63^ JMJD6 is closely correlated with lymph node involvement, distant metastases and more aggressive phenotypes, whereas depth of invasion is not correlated with the expression of JMJD6.^63^ Compared with patients with wild‐type JMJD6, patients with mutation, amplification, deep deletion or multiple alteration of JMJD6 have an unfavourable prognosis.^64^ Collectively, JMJD6 plays an important role in the development and progression of melanoma.

### 4.3 Oral cancer

Cancer stem cells comprise a small population of cells within a tumour and are responsible for initiation and long‐term sustenance of cancer.^65^ Cancer stem cells are considered as the root of cancer owing to their important role in tumorigenesis, tumour metastasis and tumour recurrence.^66^ JMJD6 is enriched in cancer stem cells, and knock‐down of JMJD6 suppresses the tumour sphere formation (a characteristic of cancer stem cells in human cancer cells) of tested cell lines, indicating that JMJD6 is required for the stem‐like properties of oral squamous cell carcinoma.^66^, ^67^, ^68^ Furthermore, overexpression of JMJD6 promotes cancer stem cell properties, including self‐renewal capacity, migration ability and resistance to chemotherapy.^66^, ^69^, ^70^ The expression of JMJD6 in oral squamous cell carcinoma cell lines is higher than that in precancerous cell lines, and it also positively correlates with the development of squamous cell carcinoma.^66^

Immunohistochemical staining of 18 normal human oral epithelia samples and 16 oral squamous cell carcinoma samples showed that the expression level of JMJD6 is higher in carcinoma tissues. In oral squamous cell carcinoma cases, the strong JMJD6 staining rate is 69%, while 89% normal human oral epithelia cases show weak JMJD6 staining. Therefore, JMJD6 plays a role in the development of oral cancer, in part because it serves as a molecular determinant of cancer stem cell phenotype.

### 4.4 Lung adenocarcinoma

Jumonji domain‐containing protein 6 mRNA and protein are significantly increased in human lung adenocarcinoma specimens than in corresponding non‐tumour lung tissues.^71^, ^72^ The level of JMJD6 is significantly associated with clinical parameters, such as tumour size, pathological grade, pathological tumour (pT) status, pathological node (pN) status and pleural invasion.^71^ In the overall survival of lung adenocarcinoma patients, JMJD6 plays a negative role.^71^, ^72^ Furthermore, the results of in vitro experiments indicated that acetylation of HOXB9 at lysine 27 decreases its ability to promote migration and growth of lung cancer cells in mice through suppressing the transcription of JMJD6, supporting that JMJD6 indeed acts as an oncogenic protein.^72^

### 4.5 Glioblastoma

Levels of JMJD6 mRNA and protein are significantly elevated in human glioma tissues, and are increased with tumour grade. Inhibiting JMJD6 with short hairpin RNA (shRNA) or single‐guide RNA (sgRNA) could not alter cell growth, colony formation and survival in vitro.^73^ In the orthotopic xenograft mouse model, targeting JMJD6 is of great benefits to survival, and sustained JMJD6 inhibition may provide even better anti‐tumour effects.^73^ In addition, compared with mice implanted with normal glioblastoma cells, the survival of mice bearing JMJD6‐deficient glioblastoma cells is significant improved. Therefore, these findings provide evidence that JMJD6 plays a key role in glioblastoma and may be a potential therapeutic target of glioblastoma.^73^, ^74^

### 4.6 Hepatocellular carcinoma

Knock‐down of JMJD6 reduces the migratory ability, proliferation rate and colony formation of hepatocellular carcinoma cell lines.^17^ The results of flow cytometry analyses showed that JMJD6 increases the proportion of cells in the S phase, reduces the proportion of cells in the G1 phase.^17^ Overexpression of JMJD6 reduces the apoptosis of human hepatoma‐derived cell lines.^17^ The JMJD6 expression level is significantly increased in human hepatocellular carcinoma tissues than in normal liver tissues.^17^ JMJD6 protein level in hepatocellular carcinoma is positively correlated with histological grade, and the JMJD6 mRNA level is significantly correlated with tumour grade and TNM stage.^17^ In addition, high JMJD6 expression in tumour tissues is indicative of poor prognosis.^17^

### 4.7 Colon carcinoma

Jumonji domain‐containing protein six knock‐down in colon cancer cell lines increases the percentage of cells in the G1 phase, reduces the cell population in the S phase, promotes cell apoptosis and sensitizes cells to DNA damaging agents.^16^ The results of xenograft experiments performed in mice showed that JMJD6 promotes cellular proliferation and tumour growth.^16^ Immunohistochemical staining of 90 colon carcinoma samples with paired adjacent normal tissues showed that JMJD6 protein is significantly increased in colon adenocarcinoma.^16^ High level of JMJD6 expression is correlated with increased invasiveness, poor differentiation, lymph node metastasis and advanced stage.^16^ In addition, follow‐up data showed that elevated JMJD6 is associated with worse outcomes of patients with colon carcinoma.^16^

### 4.8 Ovarian cancer

Inhibition of JMJD6 with compound 2‐(2‐(2‐hydroxybenzylidene) hydrazinyl)‐6‐methylpyrimidin‐4‐ol (termed SKLB325) inhibits cellular proliferation and migration, and promotes apoptosis of ovarian cancer cell lines.^75^ In the intraperitoneal xenograft model, JMJD6 inhibitor exhibits powerful therapeutic effects on ovarian cancer.^75^ Among 146 patients with ovarian cancer, JMJD6 is highly expressed in 61.64% of them.^75^ High expression of JMJD6 is significantly associated with age, clinical stage, pT status and pN status of the patients.^75^ In patients with serous and mucinous ovarian cancer, high level of JMJD6 indicates high incidence of disease progression and death.^75^

### 4.9 Neuroglioma

In a study of neuroglioma, neuroglioma stem cells with self‐renew and multipotential differentiation ability were isolated.^13^ The results of Western blot indicated that JMJD6 expression is increased notably in neuroglioma stem cells than in other neuroglioma cells.^13^ Furthermore, JMJD6 promotes proliferation, migration and invasion of neuroglioma stem cells.^13^

## 5 MECHANISMS OF JMJD6 IN PROMOTING CANCER DEVELOPMENT

**Figure 3:**
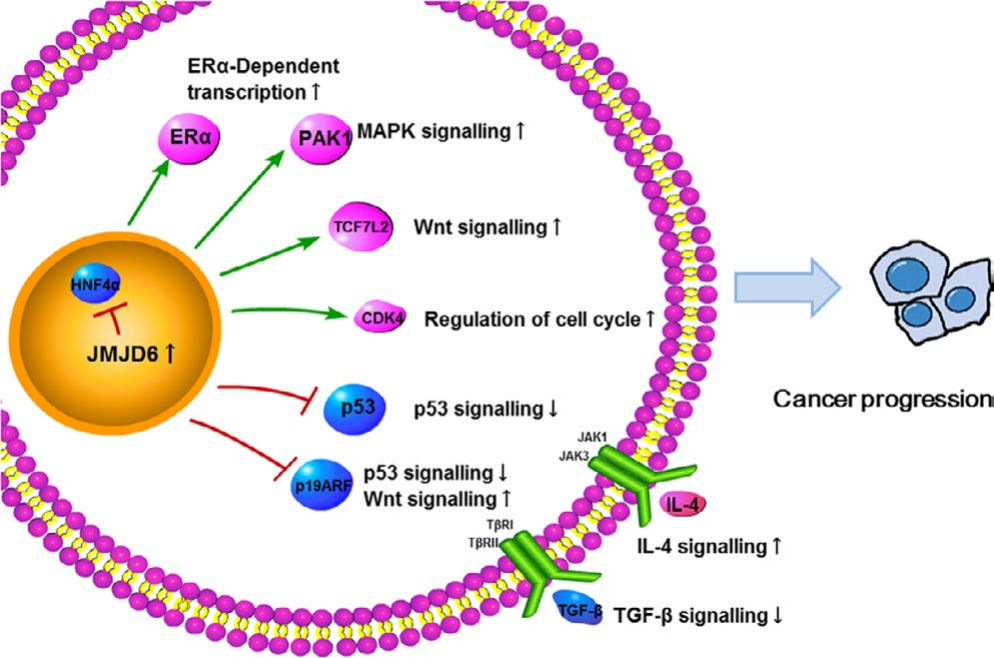
The candidate mechanisms through which JMJD6 promotes cancer progression: JMJD6 cooperates with cancer‐promoting signalling through interacting with ERα, PAK1, TCF7L2, CDK4 and IL‐4 (pink), and represses the cancer suppression signalling through regulating HNF4α, p53, p19ARF and TGF‐β (blue)

Although the mechanisms through which JMJD6 promotes cancer progression remain unclear, several mechanisms have been proposed in published studies. Here, we summarized the important candidate mechanisms (Figure 3).

### 5.1 JMJD6 regulates cancer‐related signalling

#### 5.1.1 JMJD6 downregulates p53 activity

Jumonji domain‐containing protein 6 has been reported to interact with p53 and participate in its posttranslational modification.^16^ Perturbations in p53 signalling pathways are thought to play an important role in the development of cancer, and mutations leading to loss of wild‐type p53 activity are often detected in many different types of cancer.^76^ In human colon carcinoma HCT116 cells, JMJD6 is physically associated with the tumour suppressor p53, and the C‐terminal fragment of p53 (from residues 290 to 393) is required for the binding of p53 to JMJD6. Although hydroxylation of p53 protein has not been reported before this study, it was demonstrated that JMJD6 acts as a 2‐OG‐ and Fe(II)‐dependent lysyl hydroxylase and catalyses hydroxylation of p53 on lysine 382 (K382). Furthermore, elevated expression of JMJD6 in cells leads to an increase in the amount of K382 hydroxylation of p53. HCT116 cells depleted of JMJD6 by siRNA showed increased levels of both mRNA and protein of p21 and PUMA, two well‐characterized p53 downstream target genes. JMJD6 knock‐down arrests cells in the G1 phase, induces cell apoptosis, makes cells sensitive to DNA damaging agents and represses p53‐dependent colon cell proliferation and tumour development in a p53‐dependent manner. JMJD6 negatively regulates p53 transcriptional activity through hydroxylation modification. In addition to being hydroxylated, lysine 382 of p53 can also be acetylated by the acetyl transferase p300/CBP, which has been reported to enhance the transcriptional activity of p53. However, hydroxylation of lysine 382 antagonizes p300/CBP‐mediated acetylation. Together, these findings suggested that JMJD6 catalyses hydroxylation of p53 and downregulates its transcriptional activity, thereby inhibiting the tumour suppressor function of p53.

#### 5.1.2 JMJD6 upregulates Wnt signalling

Since Wnt signalling is critical for the activity of epithelial stem cells, it is not surprising that Wnt signalling is frequently upregulated in cancer.^77^ In glioma stem cells, cignal finder cancer 10‐pathway reporter array was adopted to explore the signalling pathways involved in the association between JMJD6 and increased cell proliferation, migration and invasion.^13^ The results demonstrated that silencing of JMJD6 with JMJD6‐shRNA suppresses Wnt signalling and activates p53 signalling. Further studies measured the expression of essential molecule T‐cell factor/lymphoid enhancer factor (TCF/LEF) family protein Tcf7l2 in Wnt signalling and found that silencing of JMJD6 significantly reduces the Tcf7l2 expression.^13^ JMJD6 has also been demonstrated to interact with and depress TCF/LEF family protein Tcf7l1 (also known as Tcf3), a transcriptional repressor that inhibits transcription of Wnt target genes by recruiting Groucho‐related transcriptional corepressors.^78^, ^79^ This study then showed that JMJD6 does enhance Wnt signalling. The aa 33‐410 region of Tcf7l1 is the binding domain between Tcf7l1 and JMJD6, which is also responsible for the interaction of Tcf7l1 with Groucho.^79^ Moreover, miR‐770 binds to the 3′UTR of JMJD6 mRNA and suppresses its expression, resulting in the downregulation of WNT/β‐catenin pathway, thereby exerting anti‐tumour effect.^80^

#### 5.1.3 JMJD6 upregulates MAPK signalling

The mitogen‐activated protein kinase (MAPK) cascade is a critical pathway for human cancer cell survival, migration and resistance to drug therapy.^81^, ^82^, ^83^ RNA deep‐sequencing and bioinformatics analyses indicated that silencing of JMJD6 with siRNAs affects the alternative splicing key components of the MAPK signalling pathway, such as PAK1 (p21‐activated kinase 1), RAPGEF2 and MAP3K4.^63^ Among these, PAK1 is capable of directly phosphorylating RAF and MEK1 (mitogen‐activated protein kinase kinase 1), thereby positively regulating MAPK signalling.^63^, ^84^, ^85^ Further studies demonstrated that JMJD6 binds to PAK1 precursor messenger RNA (pre‐mRNA) and affects the alternative splicing of PAK1 through promoting exon inclusion and generation of the full‐length PAK1.^63^ Interestingly, MAPK signalling is also associated with JMJD6 expression, because of that downregulation of MAPK signalling contributes to reduced mRNA and protein levels of JMJD6.^63^ It was suggested that hyperactive MAPK signalling leads to the phosphorylation of c‐Jun, and then, activated c‐Jun transactivates JMJD6.^63^ In summary, there may be a feedforward regulatory loop between JMJD6 and the MAPK signalling pathway.

#### 5.1.4 JMJD6 suppresses Myc‐induced apoptosis

Myc expression is commonly deregulated in many cancers of different origins. Myc plays an important role in multiple cellular processes that promote survival of cancer cells.^86^, ^87^ To curb cell cycle progression in response to increased Myc, increased Myc also induces p19ARF expression, thereby leading to cell apoptosis through the activation of p53.^88^, ^89^ Furthermore, p19ARF binds with Myc and prevents Myc‐mediated tumorigenesis in a p53‐independent manner.^89^ JMJD6 cooperates with Myc to enhance tumorigenesis through suppressing Myc‐induced apoptosis.^62^ JMJD6 binds to the p19ARF promoter and demethylates Arg3 of histone H4, thereby repressing p19ARF and reducing p53 levels. Moreover, JMJD6 overexpression can induce epithelial‐mesenchymal transformation and greatly enhance tumour growth and invasion.^62^

#### 5.1.5 JMJD6 suppresses TGF‐β signalling

The transforming growth factor (TGF)‐β signalling is involved in diverse cellular processes, such as cell proliferation, differentiation, apoptosis and migration.^90^, ^91^ In the tumour development, TGF‐β signalling plays an environment‐dependent role: during the early phases, TGF‐β primarily acts as a tumour suppressor, whereas in the later phases, TGF‐β signalling promotes invasion and metastases of tumour.^92^, ^93^, ^94^ TGF‐β activates cyclin‐dependent kinase inhibitors, p15 and p21, and suppresses CDK2 (cyclin‐dependent kinase 2) and cyclin E, thereby exerting anti‐proliferative effects.^95^, ^96^ In breast cancer cell lines, the TGF‐β isoforms, especially TGF‐β2, are downregulated at both mRNA and protein levels when JMJD6 is overexpressed.^12^ Therefore, JMJD6 may mediate cellular proliferation in part by suppressing TGF‐β2. However, further studies are needed because TGF‐β2 cannot explain the cell cycle arrest in some cell lines.

### 5.2 JMJD6 interacts with Brd4

Brd4 is a well‐studied member of BET domain family of proteins which are characterized by two conserved N‐terminal bromodomains (BD1 and BD2) and an extraterminal (ET) domain.^97^ Brd4 binds to acetylated lysine residues on histone tails and other nuclear proteins through bromodomains which have modest affinity for acetylated lysine in a range of polypeptide contexts, and recruits transcriptional regulators such as positive transcription elongation factor b (P‐TEFb) via CTD (carboxyl‐terminal domain) and mediator complex to influence gene expression.^97^, ^98^, ^99^, ^100^, ^101^ Cancer‐associated genes seem to be selectively dependent on Brd4, which plays a key role in cancer development.^102^ In addition to regulating transcription, Brd4 also affects many processes like DNA damage repair and checkpoint activation or telomere homoeostasis.^102^

**Figure 4:**
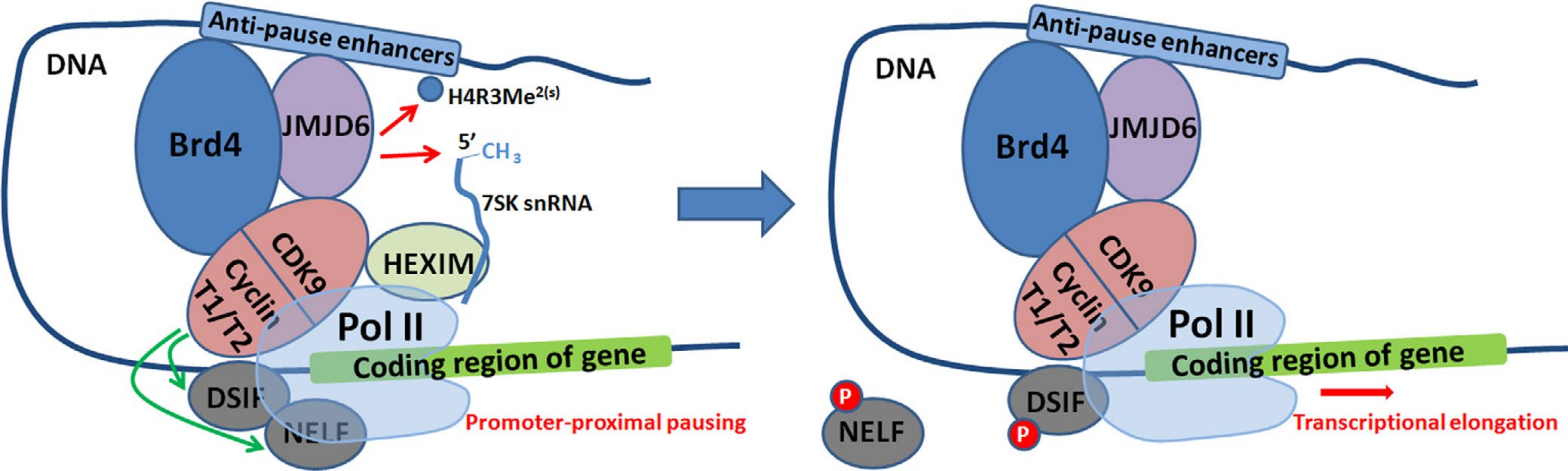
Transcriptional pause release regulation. JMJD6 and Brd4 demethylate H4R3Me^2(s)^ and the methyl cap of 7SK, and cause dismissal of the inhibitory complex 7SK snRNA/HEXIM1, thus inducing the activation of p‐TEFb. P‐TEFb is a heterodimer of cyclin‐dependent kinase 9 (CDK9) and one of cyclin subunit (cyclin T1 or cyclin T2). Activated P‐TEFb phosphorylates RNA pol II (two negative factors, DSIF and NELF, are targeted), permitting subsequent pause release for transcriptional elongation

The interaction between ET domain of Brd4 and JMJD6 has been identified using proteomic analysis in the initial studies.^103^ ET domain recognizes the α6 helix of JMJD6.^104^ As one of the ET domain interactors, JMJD6 has been shown to be critical for P‐TEFb‐independent transcriptional activation of many target genes of Brd4.^103^ A subsequent study described more detailed investigations on the interaction between Brd4 and JMJD6, and indicated that the JmjC domains and amino‐terminal of JMJD6 and the ET domain of Brd4 mediate this interaction.^11^ In particular, in the process of P‐TEFb activation and promoter‐proximal polymerase II (Pol II) pause release of a large numbers of genes, both JMJD6 and Brd4 are essential.^11^, ^105^ The pause release function of the JMJD6 and Brd4 is primarily based on their co‐binding to distal enhancers, termed anti‐pause enhancers (A‐PEs).^11^, ^73^ In terms of mechanism, JMJD6 demethylates H4R3me^2(s)^ (a repressive histone mark) and the methyl cap of 7SK snRNA (a “reader” for H4R3me^2(s)^), and causes dismissal of the inhibitory complex 7SK snRNA/HEXIM1, thus inducing the activation of P‐TEFb, and permitting subsequent pause release for transcriptional elongation (Figure 4).^11^ It is noteworthy that either JMJD6 or Brd4 can function independently in promoter‐proximal pause release for some transcription units.^11^

It has been demonstrated that JMJD6, independently of its catalytic activity, participates in the regulation of DNA damage response signalling in cells by interacting with Brd4.^106^ JMJD6 is recruited to DNA damage sites and limits the spreading of RNF168‐catalysed histone ubiquitination around DNA double‐strand breaks (DSBs).^106^ Furthermore, JMJD6 controls the subsequent recruitment of repair proteins, while the expressions of these proteins are not downregulated. Since the spreading of ubiquitination catalysed by RNF168 induces an ataxia telangiectasia‐mutated (ATM)—dependent transcriptional silencing programme in cis to DSBs, JMJD6 plays a role in limiting transcriptional silencing.^107^ This JMJD6‐mediated DNA damage response is Brd4‐dependent because Brd4 is necessary for the recruitment of JMJD6.^106^

### 5.3 JMJD6 regulates ERα‐dependent enhancer and coding gene activation

Oestrogen is a member of steroid hormone family and sustained exposure to oestrogen increases the risk of breast cancer and promotes cancer progression by stimulating proliferation of cancer cells.^108^, ^109^ The effects of oestrogen on normal and malignant breast tissues are mainly mediated by ERα, and about 70% of breast cancers are ERα positive.^110^ JMJD6 has been reported to demethylate the ERa on R260 to regulate the function of ERa.^45^ JMJD6 recruitment is required for RNA Pol II recruitment and enhancer RNA production of ERα‐bound active enhancers, leading to transcriptional pause release of cognate oestrogen target genes.^111^ Mediator complex subunit 12 (MED12) is involved in transcriptional regulation of a variety of signalling pathways, including oestrogen‐induced transcriptional activation.^112^ Mechanistically, JMJD6 specifically interacts with C‐terminus of MED12 and regulates its recruitment to ERα‐bound active enhancers, thereby affecting oestrogen‐induced transcriptional activation.^111^ Furthermore, JMJD6 is required for MED12 interaction with CARM1 (co‐activator associated arginine methyltransferase 1).^111^ CARM1 methylates the C‐terminus of MED12, which is necessary for MED12 binding with chromatin and transcriptional regulation.^113^ In mice model of breast cancer, JMJD6 knock‐down reduces the effects of oestrogen‐induced tumorigenesis, and this is dependent on the enzymatic activities of JMJD6.^111^ Therefore, JMJD6 is a critical regulator of ERα‐dependent enhancer and coding gene activation through modulating the recruitment of MED12.

### 5.4 JMJD6 suppresses HNF4α expression

A growing number of studies have shown that the expression of hepatocyte nuclear factor 4α (HNF4α) is reduced in cancers of multiple organs that normally express HNF4α.^114^ HNF4α is a member of the nuclear receptor superfamily and participates in regulating epithelial junctions, cellular metabolism, differentiation and proliferation of liver and intestinal epithelial cells.^115^ It has been demonstrated that downregulation of HNF4α promotes tumorigenesis in liver and colon, and reexpression of HNF4α represses cancer progression.^114^ Arginine methylation level in hepatocytes is mainly controlled by the activity of the protein arginine methyl transferase (PRMT) PRMT1 and the demethylase JMJD6.^116^ PRMT1 directly upregulates the expression of HNF4α through arginine methylation at the HNF4α promoter, whereas JMJD6 demethylates the HNF4α promoter and suppresses its expression.^116^ In human hepatocellular cancer specimens, a strong association between arginine methylation and HNF4α level has been observed.^116^ Therefore, in hepatocytes, PRMT1 and JMJD6 reciprocal regulate arginine methylation level and control HNF4α activity, which may be associated with the development of liver cancer.^116^ In addition, loss of arginine methylation and downregulation of HNF4α expression may contribute to alcohol‐associated liver cancer.^116^

### 5.5 JMJD6 upregulates CDK4

Cyclin‐dependent kinase 4 (CDK4) plays a key role in regulating cell cycle G1 phase progression and the G1‐S transition.^117^ Aberrant CDK4 expression may result in increased proliferation, which is frequently observed in many types of cancer, including breast cancer, hepatocellular cancer and melanoma.^118^, ^119^, ^120^, ^121^ JMJD6 promotes CDK4 expression by suppressing H4R3me^2(s)^ binding on the CDK4 promoter.^17^ Furthermore, JMJD6 interacts with p300/CBP‐associated factor (PCAF), a member of the GCN5‐related N‐acetyl transferase (GNAT) family of protein acetyltransferases, to regulate the histone modifications on the CDK4 promoter.^17^ The evidence suggesting that CDK4 is a necessary downstream effector of JMJD6 in regulating hepatoma cell proliferation also comes from the observations that the ability of JMJD6 to promote cancer cell proliferation can be abolished by inhibiting CDK4.^17^ Taken together, JMJD6 may promote hepatocellular cancer progression by targeting CDK4.

### 5.6 JMJD6 regulates angiogenesis

**Figure 5:**
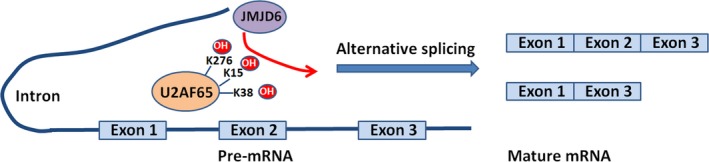
JMJD6 participates in pre‐mRNA splicing regulation through mediating lysine hydroxylation of SR protein and SR‐like splicing factors. For example: JMJD6 hydroxylates the lysine of U2AF65 (K15, 38 and 276), thus regulating alternative splicing

Abnormal splicing variants may contribute to the development of cancer in humans. It was suggested that JMJD6 regulates the splicing of vascular endothelial growth factor receptor 1 (Flt1) and controls angiogenic sprouting. Downregulation of JMJD6 alters the splicing of Flt1 and increases the levels of its soluble form which binds to vascular endothelial growth factors (VEGF) and placental growth factor, thus inhibiting angiogenesis.^50^, ^122^, ^123^, ^124^ The role of JMJD6 in splicing regulation may be achieved by its interaction with RS domains (domains rich in alternating arginine and serine residues) of serine and arginine‐rich (SR) proteins and SR‐like splicing factors, especially the splicing factor U2AF65 (Figure 5).^23^ JMJD6 interacts with U2AF65 that binds to Flt1 mRNA.^50^ More recently, it was reported that JMJD6 and U2AF65 directly bind to pre‐mRNA and coregulate a large set of alternative splicing events.^125^

### 5.7 JMJD6 acts as a tyrosine kinase and promotes autophagy

In a recent study of triple‐negative breast cancer, JMJD6 has been shown to have intrinsic tyrosine kinase activity. JMJD6 phosphorylates Y39 of histone H2A.X (H2A.X^Y39ph^) using ATP and GTP as phosphate donors. It has been reported that phosphorylation of Y39 positively regulates DNA damage response and is related to cancer progression.^126^ Not surprisingly, phosphorylation of Y39 is increased in various cancer cell lines and is associated with histological grade, tumour size and stage and survival of patients.^126^ Increased JMJD6 and H2A.X^Y39ph^ promote autophagy and triple‐negative breast cancer growth by modulating the expression of autophagy‐related proteins (ATG), including ATG5, ATG7, ATG12 and ATG13.^10^ Notably, simultaneous blocking tyrosine kinase activity of JMJD6 and autophagy is more effective in reducing the growth of triple‐negative breast cancer in mice than blocking tyrosine kinase activity of JMJD6 or autophagy alone.^10^ Therefore, the JMJD6‐H2A.X^Y39ph^ axis promotes the growth of triple‐negative breast cancer through the autophagy pathway.

### 5.8 JMJD6 induces IL4 transcription and maintains cancer stemness properties

Interleukin‐4 (IL‐4) is a multifunctional cytokine that can facilitate tumour growth and metastasis.^127^ In various types of cancer, IL‐4 is overexpressed, including colon cancer, pancreatic cancer and prostate cancer.^127^, ^128^, ^129^ In a study of oral squamous cell carcinoma, JMJD6 was found to be associated with cancer stem cell phenotype.^66^ In oral squamous cell carcinoma cell lines (SCC9/TNF and UM17b), knock‐down of JMJD6 suppresses IL‐4 mRNA expression.^66^ Subsequently, chromatin immunoprecipitation assay suggested that JMJD6 binds to the promoter of IL4.^66^ Furthermore, the addition of recombinant human IL‐4 to the JMJD6‐knock‐down oral squamous cell carcinoma cells rescues the stem‐like properties of cancer stem cells, while IL4 neutralizing suppresses the stem‐like properties.^66^ Therefore, JMJD6 induces IL4 transcription by binding to its promoter and acts as a regulator of oral cancer stem cell phenotype.

### 5.9 JMJD6 alters HOTAIR expression

The long non‐coding RNA HOX transcript antisense intergenic RNA (HOTAIR) is a key regulator of chromatin dynamics and is dysregulated in a variety of cancers.^130^, ^131^, ^132^ Overexpression of HOTAIR in epithelial cancer cells induces genome‐wide retargeting of polycomb repressive complex 2 (PRC2) and leads to altered histone methylation and gene expression profiles, resulting in tumour initiation and progression.^130^ JMJD6 may alter HOTAIR expression in a tumour‐specific manner.^133^ JMJD6 physically binds upstream of the HOTAIR transcription start site (−123 to −103 bp), and this process is independent of enzymatic activity of JMJD6.^133^ Inhibition of JMJD6 activity may reduce HOTAIR level, thus reducing tumour growth and improving the prognosis of breast cancer patients.

### 5.10 HOXB9 targets JMJD6

As a member of homeobox superfamily, homeobox‐containing B9 (HOXB9) has been demonstrated to function in embryonic development and human cancer progression.^134^, ^135^ In contrast to the wild‐type HOXB9, HOXB9 acetylated at lysine 27 decreases its ability to promote the migration and growth of lung cancer cells in mice through direct occupying the promoter of its target gene JMJD6 and suppressing the transcription of JMJD6.^72^

## 6 CONCLUSIONS

The discovery of JMJD6 as arginine demethylase, lysyl hydroxylase and tyrosine kinase of histone suggests that the protein plays a role in chromatin configuration and epigenetic regulation. Loss‐of‐function studies in knockout mice have shown that JMJD6 plays an important role in embryogenesis and tissue differentiation. In addition to gene mutations, epigenetic modifications can also disrupt gene expression and cause malignant cell transformation. Not surprisingly, JMJD6 has been demonstrated to be upregulated in a wide spectrum of human cancers, and the enzymatic activities of JMJD6 have been shown to be related to its role in cancer. Although precise mechanisms by which JMJD6 promotes tumorigenesis and tumour progression have not been elucidated, it has been well established that interaction of JMJD6 with other cancer‐related signalling pathways is one of the underlying mechanisms. Furthermore, it has been demonstrated that JMJD6 is involved in resistance to chemotherapy, such as doxorubicin, methotrexate and etoposide. By virtue of its important role in cancer, JMJD6 stands as an attractive therapeutic target. We hypothesize that inhibition of JMJD6 as a monotherapy or in combination with other anti‐tumour drugs may produce good anti‐tumour effects in human cancer.

Overall, considering the role of JMJD6 in cancer progression, we believe that targeting JMJD6 is a potential strategy for developing novel therapeutics for cancer management. However, a large number of preclinical and clinical experiments are needed to verify the effectiveness of JMJD6 inhibition in cancer therapy. Continued efforts to elucidate the physiological functions of JMJD6 and the mechanisms by which JMJD6 promotes cancer progression are also critical.

## CONFLICT OF INTEREST

The authors declare no competing financial interests.

## AUTHOR CONTRIBUTIONS

JY and SC curated the data and involved in formal analysis and writing—original draft. YY involved in formal analysis and writing—review and editing. XM wrote—original draft and wrote—review and editing. YY and BS wrote—review and editing. SY wrote—review and editing and involved in figure drawing. YW conceptualized and supervised the study. XW conceptualized, supervised, validated and wrote—review editing. All authors read and approved the final manuscript.

## Data Availability

The data that support the findings of this study are available from the corresponding author upon reasonable request.
